# The Role of Circular RNA CDR1as/ciRS-7 in Regulating Tumor Microenvironment: A Pan-Cancer Analysis

**DOI:** 10.3390/biom9090429

**Published:** 2019-08-30

**Authors:** Yutian Zou, Shaoquan Zheng, Xinpei Deng, Anli Yang, Xinhua Xie, Hailin Tang, Xiaoming Xie

**Affiliations:** 1 Department of Breast Oncology, Sun Yat-sen University Cancer Center, State Key Laboratory of Oncology in South China, Collaborative Innovation Center for Cancer Medicine, 651 East Dongfeng Road, Guangzhou 510060, China; 2 School of Medicine, Sun Yat-sen University, Guangzhou 510080, China

**Keywords:** circular RNA, CDR1as, CiRS-7, cancer, tumor microenvironment

## Abstract

Circular RNA CDR1as/ciRS-7 functions as an oncogenic regulator in various cancers. However, there has been a lack of systematic and comprehensive analysis to further elucidate its underlying role in cancer. In the current study, we firstly performed a bioinformatics analysis of CDR1as among 868 cancer samples by using RNA-seq datasets of the MiOncoCirc database. Gene ontology (GO), Kyoto Encyclopedia of Genes and Genomes (KEGG), gene set enrichment analysis (GSEA), CIBERSORT, Estimating the Proportion of Immune and Cancer cells (EPIC), and the MAlignant Tumors using Expression data (ESTIMATE) algorithm were applied to investigate the underlying functions and pathways. Functional enrichment analysis suggested that CDR1as has roles associated with angiogenesis, extracellular matrix (ECM) organization, integrin binding, and collagen binding. Moreover, pathway analysis indicated that it may regulate the TGF-β signaling pathway and ECM-receptor interaction. Therefore, we used CIBERSORT, EPIC, and the ESTIMATE algorithm to investigate the association between CDR1as expression and the tumor microenvironment. Our data strongly suggest that CDR1as may play a specific role in immune and stromal cell infiltration in tumor tissue, especially those of CD8+ T cells, activated NK cells, M2 macrophages, cancer-associated fibroblasts (CAFs) and endothelial cells. Generally, systematic and comprehensive analyses of CDR1as were conducted to shed light on its underlying pro-cancerous mechanism. CDR1as regulates the TGF-β signaling pathway and ECM-receptor interaction to serve as a mediator in alteration of the tumor microenvironment.

## 1. Introduction

According to statistics from the World Health Organization, cancer is regarded as the leading cause of death worldwide, and continues to remain an issue in the 21st century [1]. As presented in the global cancer statistics, 18,078,957 new cancer cases and 9,555,027 deaths due to various cancers were estimated for 2018 [2]. Despite the constant effort and progress made in oncological research (surgery, chemotherapy, radiotherapy, targeted therapy, etc.), there are many types of tumors that still remain incurable because of the lack of effective therapeutic targets. Therefore, it is imperative to identify novel diagnostic biomarkers and develop more efficient therapeutic molecular targets in cancer.

Circular RNAs (circRNAs) are a type of endogenous noncoding RNA which have recently attracted enormous attention and become a hot research topic in the field of biomedicine [3]. Being widely expressed and present in mammalian tissues, circRNAs used to be considered as byproducts of incorrect splicing in cells. Compared to linear RNAs, circRNAs are more stable, and do not possess either a 5′-cup or 3′-poly A tail [4]. CircRNAs are versatile regulators of the cellular activities underlying various biological functions, including as a miRNA-binding sponge, RNA-binding protein regulators, protein translation templates, among others [5]. In a recent study, the newly developed exome capture RNA sequencing technology was employed to detect and identify circRNAs in over 2000 cancer samples to build the comprehensive database MiOncoCirc [6]. This database, with its large sample size, is an invaluable resource for the analysis of circRNAs across all cancer types.

Derived from cerebellar degeneration-related protein 1 antisense transcript (CDR1AS), CDR1as is the best-known circRNA, and has been uncovered as a regulator of cellular processes containing over 70 conventional binding sites for miR-7 [7,8]. On the basis of the competing endogenous RNA (ceRNA) hypothesis, CDR1as serves as a negative regulator of miR-7 and exerts an influence on the expression of multiple key genes [9,10]. For this reason, CDR1as is also termed ciRS-7, which stands for a circular RNA sponge for miR-7. Since miR-7 has been widely studied and previously determined to be a tumor suppressor in diverse cancers, CDR1as has rapidly become a hotspot of research in cancer [11,12,13,14,15]. For instance, CDR1as acts as a miR-7 sponge to promote colorectal cancer progression through regulating EGFR-RAF1 activity [16]. It can also act as an inhibitor of another miRNA, miR-1299, to facilitate breast cell carcinoma growth and metastasis via targeting of MMP expression [17]. Hence, CDR1as is poised to become a novel diagnostic biomarker and specific therapeutic target for a diversity of cancers. However, there is a lack of systematic and comprehensive analysis of CDR1as to further elucidate its biological function in cancer.

In the current study, we first performed a comprehensive bioinformatics analysis of CDR1as using RNA-seq datasets of the MiOncoCirc database. We mapped the expression spectrum of CDR1as in different tumors and metastasis sites. By conducting a series of bioinformatics analyses, CDR1as was found to have functions associated with angiogenesis, extracellular matrix (ECM) organization, integrin binding, and collagen binding. Moreover, pathway analysis indicated that it may activate the TGF-β signaling pathway and ECM-receptor interaction. We then used CIBERSORT, Estimating the Proportion of Immune and Cancer cells (EPIC), and the MAlignant Tumors using Expression data (ESTIMATE) algorithm to investigate the association between CDR1as expression and the tumor microenvironment. Our data strongly suggest that CDR1as may play a specific role in immune and stromal cell infiltration in tumor tissue, especially those of CD8+ T cells, activated NK cells, M2 macrophages, CAFs and endothelial cells. The systematic and comprehensive analysis of CDR1as was conducted to shed light on its underlying pro-cancerous mechanism in altering the tumor microenvironment.

## 2. Methods

### 2.1. Database Available and Ethics Approval

CircRNA-seq and linear mRNA expression data were downloaded from the MiOncoCirc database (https://nguyenjoshvo.github.io/) [6]. Clinical information such as cancer type, biopsy site, and metastasis site were also obtained from the MiOncoCirc database portal. This study was approved by the Ethics Committee of Sun Yat-Sen University Cancer Center Health Authority (ethical code number: GZR2017-163) and was performed according to the ethical standards of the Declaration of Helsinki

### 2.2. Identification of Differentially Expressed Genes and Co-Expression Genes

The R package ′limma’ was used to perform data analysis. We set the fold change to >2 and adjusted *P* < 0.05 as the cutoff value to identify differentially expressed genes. Heatmap was generated using R software version 3.5.0 (Free Software Foundation, Boston, MA, USA). Linear regression analysis was carried out to screen for co-expressed genes using the R packages “corrplot” and “Hmisc”. Spearman correlation analysis was used as the statistical approach.

### 2.3. Functional Enrichment Analysis

Both Gene Ontology (GO) functional enrichment analysis and Kyoto Encyclopedia of Genes and Genomes (KEGG) pathway enrichment analysis were performed utilizing the “GOplot” package in R software. FDR < 0.05 was set as the threshold. Gene set enrichment analysis (GSEA) was applied to identify the significantly enriched pathways between the CDR1as high and low expression group using the GSEA 3.0 desktop application.

### 2.4. Construction of the Protein–Protein Interaction (PPI) Network and ceRNA Network

The protein–protein interaction (PPI) network was built via the STRING online website (https://string-db.org/) and Cytoscape software version 3.7.1 (The Cytoscape Consortium, San Diego, CA, USA). We only included networks with no fewer than 10 nodes for further analysis. The Molecular COmplex DEtection (MCODE) application was then employed to find clustered molecules in the global networks. The ceRNA network was constructed via Cytoscape software version 3.7.1. Prediction of the miRNA binding sites of CDR1as was conducted using the Circular RNA Interactome website (https://circinteractome.nia.nih.gov/). The TargetScan algorithm was used to identify the potential target oncogenes of miRNAs (http://www.targetscan.org/).

### 2.5. Evaluation of Tumor Microenvironment

For the prediction of tumor infiltrating lymphocytes (TILs), the expression matrices were uploaded to CIBERSORT (https://cibersort.stanford.edu/) and calculated according to the LM22 signature with 1000 permutations [18]. The valid samples were selected using *P* < 0.05. For the estimation of cancer-associated fibroblasts (CAFs) and endothelial cell infiltration, the EPIC algorithm was employed to calculate certain infiltrating stromal cells [19]. Samples without a full convergence of optimization were discarded. To predict total immune and stromal infiltration, the MAlignant Tumors using Expression data (ESTIMATE) algorithm was used to calculate the immune score and stromal score, as described in reference [20]. The ESTIMATE score was the combination of the above two scores, and reflected the tumor purity.

## 3. Results

### 3.1. The Expression Level of CDR1as in Different Types of Human Cancers

**Figure 1:**
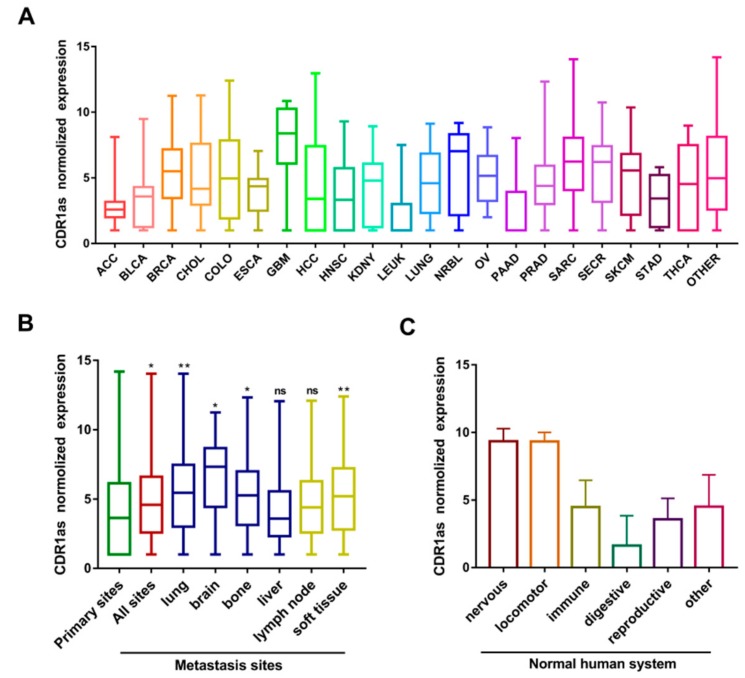
Expression spectrum of CDR1as in different tissues according to MiOncoCirc database. (**A**) Expression level of CDR1as in different types of cancer. (**B)** Expression level of CDR1as in different biopsy sites. (**C**) Expression level of CDR1as in different normal human systems.

To map the expression spectrum of CDR1as in tumor and normal human organs, we analyzed its profile in different cancers and systems using the MiOncoCirc database. In general, RNA-seq datasets of 1200 samples from 28 different cancer tissues were available and processed from the database. As shown in Figure 1A, the expression level of CDR1as was relatively higher in glioblastoma multiforme (GBM), neuroblastoma (NRBL), sarcoma (SARC), secretory cancer (SECR), breast cancer (BRCA), and melanoma (SKCM). To investigate the relationship between CDR1as expression and metastasis, we divided the samples into groups based on the metastasis site. The CDR1as expression level increased in metastasis sites compared to primary sites. It was also positively correlated with lung, brain, bone, and soft tissue metastasis of cancer (Figure 1B). Due to the limited sample size and representativeness of normal tissues, we compared the CDR1as profile in different human systems. The data revealed that CDR1as expression was high in the nervous system (brain and spinal cord) and locomotor system (bone), while being low in the digestive system (stomach, liver, small intestine, and colon) (Figure 1C). Moreover, the abundance of circular transcripts of CDR1as has a strong correlation with the abundance of linear transcripts of mCDR1AS, which means that the elevated expression of CDR1as could be directly explained by upregulation of the parental transcript (Supplementary Figure S1A).

### 3.2. Comparison of Gene Expression Profile and Functional Enrichment Analysis of CDR1as in Cancers

**Figure 2:**
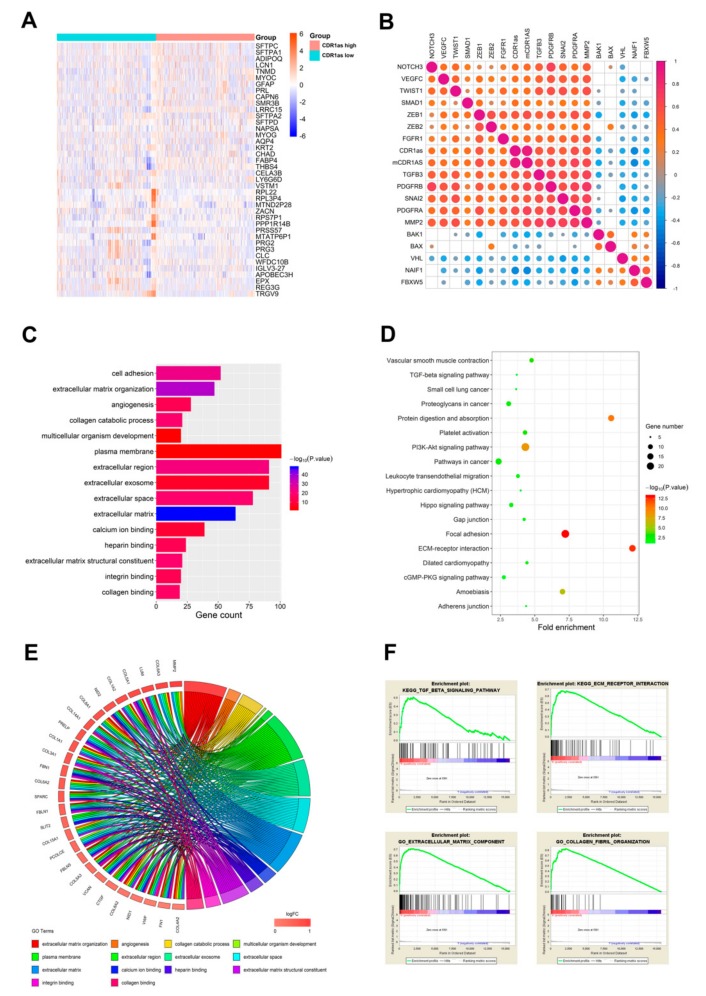
Comparison of gene expression profile and functional enrichment analysis of CDR1as in cancers. (**A**) Heatmap of the differential expressed gene of CDR1as high expression group vs. low expression group (fold change > 2, *P* < 0.05). Top 20 upregulated and downregulated genes are presented. (**B**) Matrix displays some of the selected genes correlated with CDR1as expression. The color scale and the size of the dots indicate the values of pairwise Spearman rank correlation. (**C**) Top 5 gene ontology (GO) terms in cellular component, biological process and molecular function. (**D**) All enriched pathways in Kyoto Encyclopedia of Genes and Genomes (KEGG) analysis with statistical significance. (**E**) Circular layout of GO analysis revealed the most frequent genes and enriched terms. (**F**) Gene set enrichment analysis (GSEA) showed CDR1as has a significant correlation with TGF-β signaling pathway, ECM-receptor interaction, extracellular matrix component and collagen fibril organization.

To gain insight into the correlation of gene expression profiles with CDR1as expression, we analyzed 868 cancer samples with both gene expression in FPKM and circRNA sequencing statistics from the MiOncoCirc database. We divided cases into low CDR1as expression and high CDR1as expression groups based on median CDR1as expression of all samples. Heatmap revealed differential gene expression profiles of samples belong to high versus low CDR1as expression groups (Figure 2A). In total, 365 genes were upregulated and 794 genes were downregulated in the high with respect to the low CDR1as expression group (fold change > 2, *P* < 0.05). Spearman correlation analysis of CDR1as and gene expression identified 348 positively and 177 negatively correlated genes (*r* > 0.4 or < −0.4, *P* < 0.05). Among them, we found that CDR1as expression was positively associated with a series of powerful oncogenes (*PDGFRA/B*, *MMP2*, *ZEB1/2*, *VEGFC*, *SNAI2*, etc.) and negatively correlated with a range of tumor suppressors (*BAX*, *VHL*, *BAK1*, *FBXW5*, etc.) (Figure 2B). Further functional enrichment analysis was conducted to explore the potential function of CDR1as. Altogether, 15 gene ontology (GO) terms of biological process, 15 GO terms of cellular component and 11 GO terms of molecular function were identified to be significant (FDR < 0.05). The top 5 GO terms of each part are presented in Figure 2C, including cell adhesion, angiogenesis, plasma membrane, extracellular matrix, integrin binding and collagen binding. The circular layout of GO analysis revealed the most frequent genes and enriched terms (Figure 2E). Correlation analysis verified the strong positive correlation between CDR1as expression and these genes (Supplementary Figure S1B). Additionally, Kyoto Encyclopedia of Genes and Genomes (KEGG) analysis showed that each of the enriched 18 pathways included the TGF-β signaling pathway and ECM-receptor interaction (Figure 2D). To further investigate the biological functions of CDR1as, we conducted a gene set enrichment analysis (GSEA) and discovered that high expression of it significantly correlated with TGF-β signaling pathway, ECM-receptor interaction, extracellular matrix component and collagen fibril organization (Figure 2F).

### 3.3. Protein–Protein Interaction Network of the Co-Expressed Genes

**Figure 3:**
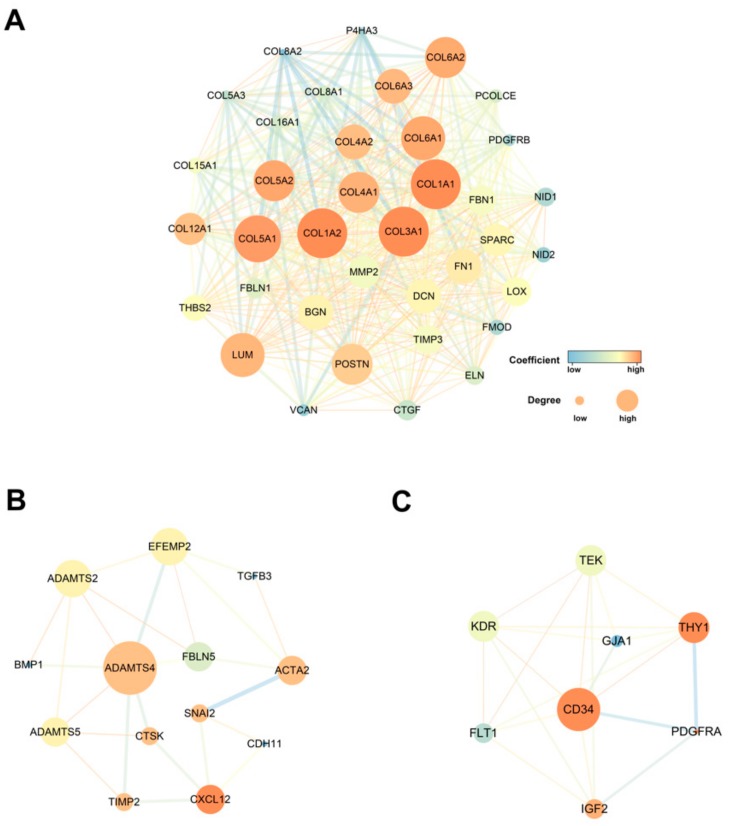
Top 3 modules of PPI networks: (**A**) COL1A1 module, (**B**) ADAMTS4 module and (**C**) CD34 module. The size of node in the PPI network reflects the number of interacting proteins with the certain protein, and the color scale indicates the clustering coefficient value of the protein.

We construct a protein–protein interaction (PPI) network utilizing the online STRING tool to achieve a better understanding of the interplay among co-expression genes. We used the Molecular COmplex DEtection (MCODE) app to discover clustered modules among the whole network. The network consisted of 18 modules containing 452 nodes and 2,529 edges. The top three significant modules were selected for further analysis (Figure 3). Among them, module 1, including 37 nodes and 511 edges, had the highest score in above clusters (Figure 3A). Module 2 (13 nodes and 24 edges) and module 3 (8 nodes and 21 edges) are also shown (Figure 3B,C). The expression of these genes in the network is highly correlated with CDR1as expression (Supplementary Table S1). Most of these genes were related to extracellular matrix formation and played a pivotal role in cancer progression.

### 3.4. Relationship between CDR1as Expression and Tumor Microenvironment

**Figure 4:**
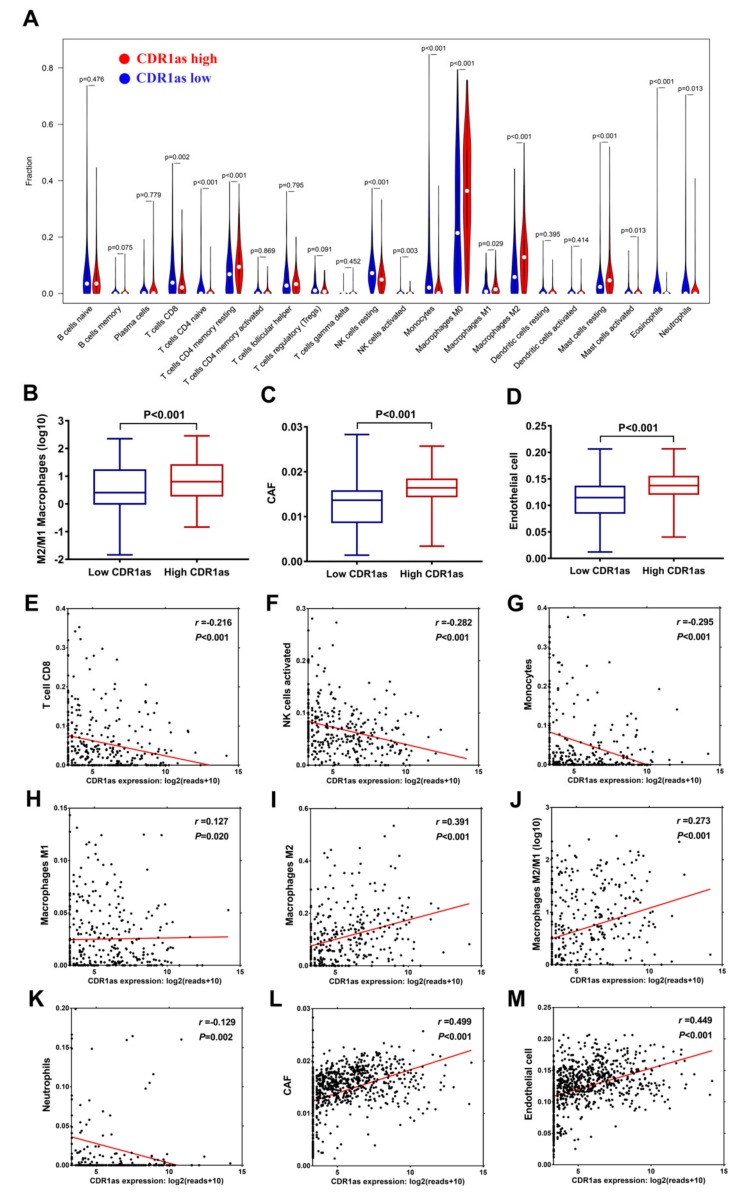
Relationship between cerebellar degeneration-related protein 1 antisense transcript (CDR1as) expression and certain immune and stromal cell infiltration evaluated by CIBERSORT and the EPIC algorithm, respectively. (**A**) Relative density of 22 types of infiltrated immune cell in CDR1as a high and low expression group. (**B**) Ratio of M2 to M1 macrophages in CDR1as a high and low expression group. (**C**–**D**) Relative density of cancer-associated fibroblasts (CAFs) and endothelial cells in CDR1as high and low expression groups. (**E**–**H**) Correlation of CDR1as expression with (**E**) CD8^+^ T cells, (**F**) activated NK cells, (**G**) monocytes, (**H**) M1 macrophages, (**I**) M2 macrophages, (**J**) ratio of M2 to M1 macrophages, (**K**) neutrophils, (**L**) CAFs, and (**M**) endothelial cells.

**Figure 5:**
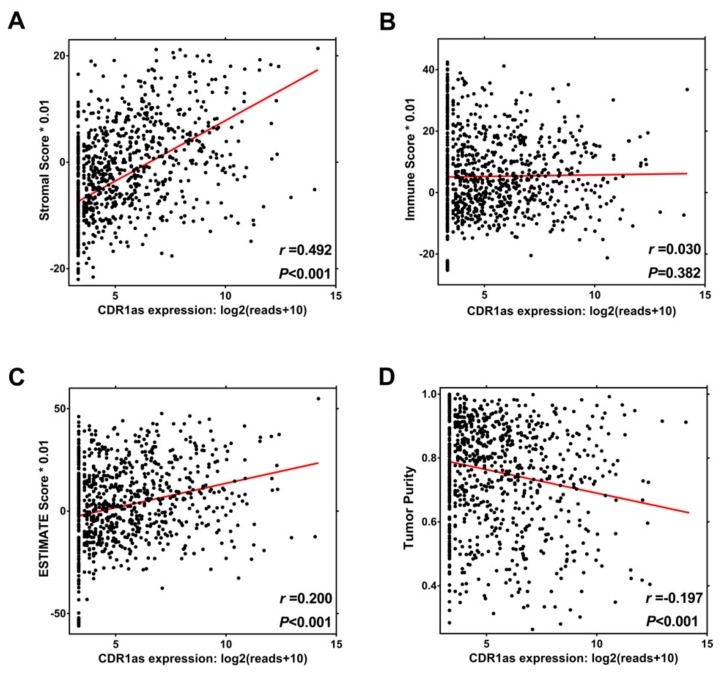
Relationship between CDR1as expression and tumor microenvironment evaluated by ESTIMATE algorithm. Correlation of CDR1as expression with (**A**) Stromal score, (**B**) Immune score, (**C**) ESTIMATE score and (**D**) Tumor purity.

As mentioned, GO analysis revealed that CDR1as has functions associated with angiogenesis, extracellular matrix, integrin binding, and collagen binding, while KEGG analysis indicated its involvement in TGF-β signaling and the ECM-receptor interaction pathway. It is well-known that stimulating the TGF-β signaling pathway and remodeling the extracellular matrix (ECM) can exert a great influence on the tumor microenvironment [21,22,23,24,25,26,27,28,29]. Therefore, we further explored whether CDR1as expression was correlated with the degree of immune and stromal infiltration in cancer. The 22 immune cell faction was calculated by CIBERSORT, and 330 valid samples were selected after filtering. The high CDR1as expression group has a lower density of CD8+ T cells, activated NK cells, M1 macrophages, M2 macrophages, monocytes, and neutrophils compared to the low CDR1as expression group (Figure 4A). It is well-acknowledged that the polarization from the antitumor M1 (classically activated) macrophage to protumor M2 (alternatively activated) macrophage phenotype is associated with tumor progression [30,31,32]. We found that the high CDR1as expression group had a higher ratio of M2 macrophage (Figure 4B). CDR1as expression was negatively correlated with CD8+ T cells (*r* = −0.216, *P* < 0.001), activated NK cells (*r* = −0.282, *P* < 0.001), monocytes (*r* = −0.295, *P* < 0.001), and neutrophils (*r* = −0.129, *P* = 0.002), and positively correlated with the M2/M1 macrophage ratio (*r* = 0.391, *P* < 0.001) (Figure 4E–K ). We then used the EPIC method to estimate the infiltration of two stromal cells: CAFs and endothelial cells. After excluding the cases without fully converged optimization, 809 samples were selected for the analysis. As shown in Figure 4C,D, the high CDR1as expression group has a higher infiltrating level of both CAFs and endothelial cells. Similarly, there were positive correlations between CDR1as expression and CAFs (*r* = 0.499, *P* < 0.001) and endothelial cells (*r* = 0.449, *P* < 0.001) (Figure 4L–M). To reveal the level of total immune and stromal infiltration, we used the MAlignant Tumors using Expression data (ESTIMATE) algorithm to calculate the immune and stromal score in each sample [20]. Sarcoma, leukemia, and gastrointestinal stromal tumors were excluded for the high and tumor-intrinsic expression of immune or stromal-related genes. CDR1as expression was positively correlated with the stromal score (*r* = 0.492, *P* < 0.001), which meant more stromal cells (fibroblasts, endothelial cells, mesenchymal stromal cells, etc.) were gathered and infiltrated in the tumor microenvironment (Figure 5A). However, we were unable to achieve a statistically significant immune score (Figure 5B). The association of CDR1as expression with the composite ESTIMATE score is shown in Figure 5C. In addition, we discovered a negative correlation between CDR1as expression with tumor purity (*r* = −0.197, *P* < 0.001) (Figure 5D). These results strongly suggested that CDR1as plays a specific role in immune and stromal infiltration in tumor tissue, especially those of CD8+ T cells, activated NK cells, M2 macrophages, CAFs, and endothelial cells.

### 3.5. ceRNA Network Revealed the Mechanism of CDR1as in Regulating the Tumor Microenvironment

**Figure 6:**
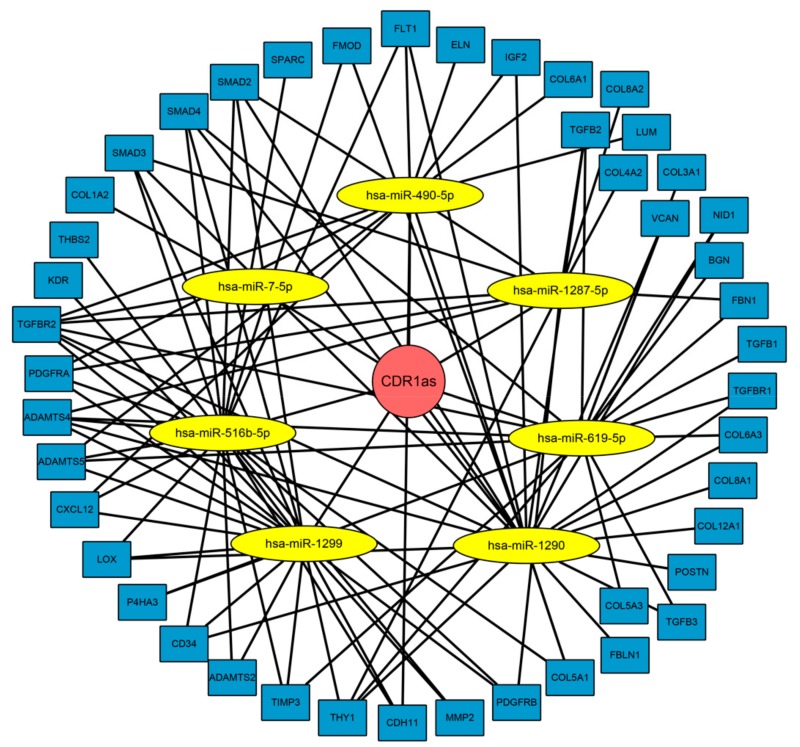
A competing endogenous RNA (ceRNA) network was constructed to illustrate the biological mechanism of CDR1as, which serves as a sponge for multiple miRNAs to relieve silencing for several key genes and regulate the tumor microenvironment.

Considering that CDR1as can bind to certain miRNAs as a miRNA sponge, we next explored whether it can act as a ceRNA in regulating several key genes which are critical mediators in the tumor microenvironment. According to the prediction of Circular RNA Interactome, CDR1as has 25 miRNA binding sites, from which we chose miRNAs with more than five binding sites on CDR1as for further analysis. Seven miRNAs (miR-7-5p, miR-490-5p, miR-516b-5p, miR-619-5p, miR-1287-5p, miR-1290, and miR-1299) met the criteria, and two of them (miR-7-5p and miR-1299) have been demonstrated to have direct interactions with CDR1as [17,33]. We then used the TargetScan algorithm to identify the potential target oncogenes of these miRNAs and found that most target genes are important genes in the TGF-β signaling pathway and ECM–receptor interaction. A ceRNA network was constructed as shown in Figure 6 to illustrate the biological mechanism of CDR1as, which serves as a sponge for multiple miRNAs to relieve silencing of several key genes and regulate the tumor microenvironment.

## 4. Discussion

As new rising stars of noncoding RNAs, circRNAs are an intriguing class of RNA because of their closed loop structure, high stability, and versatile functions in gene modulation [34]. Thanks to the rapid development in high-throughput sequencing technology and bioinformatics, numerous circRNAs have been discovered as critical regulators of various biological progress in recent years. Emerging evidence shows that circRNAs act as oncogenic stimuli or tumor suppressors via regulating protein expression in multiple cancers. For example, circCTIC1 promotes the proliferation of tumor-initiating cells in colon cancer [35]. In our previous studies, circKIF4A and circRAD18 were also discovered to be oncogenic regulators in triple-negative breast cancer [36,37].

Undoubtedly, CDR1as/ciRS-7 is a celebrity in the family of circRNA, containing over 70 conventional binding sites for miR-7 and functioning in a diversity of biological functions [11,12]. CDR1as/ciRS-7 functions as a pro-cancerous regulator by serving as a miRNA sponge in different types of cancer. For example, CDR1as was found to be a promising biomarker and target in colorectal cancer [16]. CDR1as promotes tumor growth and metastasis by binding miR-7 in esophageal and laryngeal squamous cell carcinoma [38,39]. In breast cancer, CDR1as was also discovered to have tumor-promoting functions [17]. CDR1as can also increase cell surface PD-L1 protein level in colon cancer cells [40]. However, all of these studies only investigated its function of promoting proliferation and invasion of cancer cells though interaction with miR-7/1299. There is a lack of systematic and comprehensive analysis to further elucidate its underlying pivotal role in cancer. Owing to the establishment of the MiOncoCirc database using exome capture RNA sequencing technology, circRNA-seq data of over 2000 tissues and the mRNA profile of 868 cancer samples were available for further bioinformatics analysis [6].

The tumor microenvironment is regarded as a protective shelter for cancer cells to evade immune surveillance and drug intervention [41,42]. Several components participate in the formation of the tumor microenvironment, such as fibroblasts, endothelial cells, mesenchymal stem cells, neutrophils, and macrophages, which exert a great influence on cancer growth and metastasis [43,44,45]. Stromal infiltration alters the tumor microenvironment and facilitates the initiation, proliferation, metastasis, and chemoresistance of solid tumors [46]. For instance, cancer-associated fibroblasts (CAFs) and mesenchymal stem cells (MSCs) nourish cancer cells by secreting growth factors such as the fibroblast growth factor (FGF) and hepatocyte growth factor (HGF) [47]. As is well-established, the TGF-β signaling pathway and remodeling of the extracellular matrix (ECM) play an essential role in the regulation of components of the tumor microenvironment leading to tumor progression [22,23,24,25,26,27,28,29]. The development of CIBERSORT, EPIC, and the ESTIMATE algorithm has enabled us to predict the immune and stromal infiltration status by calculating the corresponding score in tumor samples with great accuracy [18,19,20].

In the current study, we took full advantage of the MiOncoCirc database and conducted bioinformatics analysis of CDR1as using RNA-seq datasets. The expression spectrum of CDR1as in different tumors and metastasis sites was mapped. In functional enrichment analysis, CDR1as was found to have functions associated with angiogenesis, extracellular matrix organization, integrin binding, and collagen binding. Additionally, pathway analysis indicated that it may activate the TGF-β signaling pathway and ECM-receptor interaction, which is consistent with a previous report [48]. The top three modules in the protein–protein interaction network were related to extracellular matrix formation and interaction. Highly interrelated nodes, such as *COL1A1*, *COL1A2*, *POSTN*, *ADAMTS4*, and *CD34*, have been reported to regulate the tumor microenvironment and promote the growth, angiogenesis, and invasion of malignancies [49,50,51,52]. We then used the CIBERSORT, EPIC, and ESTIMATE methods to evaluate the tumor microenvironment status of each sample and found that CDR1as may play a specific role in immune and stromal infiltration in tumor tissue, especially where CD8+ T cells, activated NK cells, M2 macrophages, CAFs, and endothelial cells are involved. Finally, a ceRNA network was constructed to illustrate the biological mechanism of CDR1as, which serves as a sponge for multiple miRNAs to influence the expression of several key genes to regulate the tumor microenvironment.

In conclusion, we conducted a systematic and comprehensive analysis of CDR1as to shed light on its underlying pro-cancerous mechanism in altering the tumor microenvironment. CDR1as regulates the TGF-β signaling pathway and ECM-receptor interaction to serve as a mediator in alteration of the tumor microenvironment.

## Supplementary Materials

The following are available online at https://www.mdpi.com/2218-273X/9/9/429/s1, Figure S1: Correlation between circular RNA CDR1as and linear mRNA expression, Table S1: Linear regression analysis between CDR1as expression and genes in the Protein-protein interaction network.

## Abbreviations

ACC, Adrenocortical carcinoma; BLCA, Bladder urothelial carcinoma; BRCA, Breast invasive carcinoma; CHOL, Cholangiocarcinoma; COLO, Colorectal cancer; ESCA, Esophageal carcinoma; GBM, Glioblastoma; HCC, Hepatocellular carcinoma; HNSC, Head and neck squamous cell carcinoma; KDNY, Kidney cancer; LEUK, leukemia; LUNG, Lung cancer; NRBL, neuroblastoma; OV, Ovarian cancer; PAAD, Pancreatic adenocarcinoma; PRAD, Prostate adenocarcinoma; SARC, Sarcoma; SECR, Secretory cancer; SKCM, Skin cutaneous melanoma; STAD, Stomach adenocarcinoma; THCA, Thyroid carcinoma; OTHER, including atypical teratoid rhabdoid tumor, Langerhans cell histiocytosis, lower grade glioma, lymphoma, rhabdomyosarcoma, testicular germ cell tumor, and thymomas.

ceRNAs: CAF, cancer-associated fibroblasts; ceRNAs: competitive endogenous RNAs; circRNAs: circular RNAs; ECM, extracellular matrix; FDR: false discovery rate; GO: Gene Ontology; GSEA: Gene set enrichment analysis; KEGG: Kyoto Encyclopedia of Genes and Genomes; miRNAs: microRNAs; TGF-β: transforming growth factor-β.
