# Supplementary material for: The Role of Circular RNA CDR1as/ciRS-7 in Regulating Tumor Microenvironment: A Pan-Cancer Analysis
Source: Biomolecules. 2019 Aug 30;9(9):429. doi: 10.3390/biom9090429 (PMC6770779; doi:10.3390/biom9090429)
Supplement: Supplementary file 1 [file biomolecules-09-00429-s001.zip › Supplementary File(s)/Supplementary Table.docx]

**Supplemental Table 1. Linear regression analysis between CDR1as expression and genes in the Protein-protein interaction network.**

| Gene | *r* | *P* value |
| --- | --- | --- |
| PDGFRA | 0.573362658 | 1.64E-73 |
| MMP2 | 0.556303579 | 3.98E-68 |
| BGN | 0.550796699 | 1.87E-66 |
| CDH11 | 0.550001309 | 3.25E-66 |
| DCN | 0.548993841 | 6.50E-66 |
| COL6A3 | 0.545527827 | 6.97E-65 |
| TGFB3 | 0.542146999 | 6.87E-64 |
| LUM | 0.541816848 | 8.58E-64 |
| COL5A1 | 0.535493995 | 5.75E-62 |
| COL1A2 | 0.532132456 | 5.18E-61 |
| PDGFRB | 0.531992299 | 5.68E-61 |
| COL12A1 | 0.531393622 | 8.38E-61 |
| NID2 | 0.523090205 | 1.70E-58 |
| COL16A1 | 0.521511304 | 4.60E-58 |
| COL8A1 | 0.520908846 | 6.72E-58 |
| COL5A2 | 0.517618858 | 5.22E-57 |
| THY1 | 0.514727962 | 3.11E-56 |
| COL1A1 | 0.50867651 | 1.23E-54 |
| COL3A1 | 0.50756153 | 2.40E-54 |
| FBN1 | 0.507015566 | 3.33E-54 |
| EFEMP2 | 0.503949976 | 2.06E-53 |
| TIMP3 | 0.502744658 | 4.21E-53 |
| COL6A2 | 0.501949128 | 6.72E-53 |
| SPARC | 0.501897032 | 6.93E-53 |
| COL6A1 | 0.494244355 | 5.91E-51 |
| FBLN1 | 0.48575316 | 7.19E-49 |
| SNAI2 | 0.483746264 | 2.19E-48 |
| POSTN | 0.473157304 | 6.98E-46 |
| COL15A1 | 0.466517325 | 2.34E-44 |
| TEK | 0.465296477 | 4.43E-44 |
| PCOLCE | 0.463231133 | 1.29E-43 |
| ACTA2 | 0.46063424 | 4.93E-43 |
| THBS2 | 0.45846672 | 1.49E-42 |
| CTSK | 0.458425628 | 1.52E-42 |
| FBLN5 | 0.456184697 | 4.75E-42 |
| ADAMTS2 | 0.455636668 | 6.26E-42 |
| COL5A3 | 0.455020085 | 8.55E-42 |
| ELN | 0.448638469 | 2.05E-40 |
| VCAN | 0.443161739 | 2.98E-39 |
| CTGF | 0.438278007 | 3.11E-38 |
| COL8A2 | 0.435433 | 1.20E-37 |
| COL4A1 | 0.432807754 | 4.11E-37 |
| NID1 | 0.428015061 | 3.79E-36 |
| FMOD | 0.424552847 | 1.85E-35 |
| CD34 | 0.423710312 | 2.71E-35 |
| IGF2 | 0.422213018 | 5.33E-35 |
| P4HA3 | 0.42097933 | 9.30E-35 |
| FLT1 | 0.417981937 | 3.55E-34 |
| TIMP2 | 0.416568176 | 6.65E-34 |
| LOX | 0.413144707 | 3.00E-33 |
| CXCL12 | 0.413020912 | 3.17E-33 |
| ADAMTS5 | 0.411482796 | 6.20E-33 |
| BMP1 | 0.411362929 | 6.53E-33 |
| FN1 | 0.410880622 | 8.05E-33 |
| GJA1 | 0.409481962 | 1.48E-32 |
| ADAMTS4 | 0.409460629 | 1.49E-32 |
| KDR | 0.40716026 | 4.01E-32 |
| COL4A2 | 0.405741922 | 7.35E-32 |
| PDGFRA | 0.573362658 | 1.64E-73 |
| MMP2 | 0.556303579 | 3.98E-68 |
| BGN | 0.550796699 | 1.87E-66 |
| CDH11 | 0.550001309 | 3.25E-66 |
| DCN | 0.548993841 | 6.50E-66 |
| COL6A3 | 0.545527827 | 6.97E-65 |
| TGFB3 | 0.542146999 | 6.87E-64 |
| LUM | 0.541816848 | 8.58E-64 |
| COL5A1 | 0.535493995 | 5.75E-62 |
| COL1A2 | 0.532132456 | 5.18E-61 |
| PDGFRB | 0.531992299 | 5.68E-61 |
| COL12A1 | 0.531393622 | 8.38E-61 |
| NID2 | 0.523090205 | 1.70E-58 |
| COL16A1 | 0.521511304 | 4.60E-58 |
| COL8A1 | 0.520908846 | 6.72E-58 |
| COL5A2 | 0.517618858 | 5.22E-57 |
| THY1 | 0.514727962 | 3.11E-56 |
| COL1A1 | 0.50867651 | 1.23E-54 |
| COL3A1 | 0.50756153 | 2.40E-54 |
| FBN1 | 0.507015566 | 3.33E-54 |
| EFEMP2 | 0.503949976 | 2.06E-53 |
| TIMP3 | 0.502744658 | 4.21E-53 |
| COL6A2 | 0.501949128 | 6.72E-53 |
| SPARC | 0.501897032 | 6.93E-53 |
| COL6A1 | 0.494244355 | 5.91E-51 |
| FBLN1 | 0.48575316 | 7.19E-49 |
| SNAI2 | 0.483746264 | 2.19E-48 |
| POSTN | 0.473157304 | 6.98E-46 |
| COL15A1 | 0.466517325 | 2.34E-44 |
| TEK | 0.465296477 | 4.43E-44 |
| PCOLCE | 0.463231133 | 1.29E-43 |
| ACTA2 | 0.46063424 | 4.93E-43 |
| THBS2 | 0.45846672 | 1.49E-42 |
| CTSK | 0.458425628 | 1.52E-42 |
| FBLN5 | 0.456184697 | 4.75E-42 |
| ADAMTS2 | 0.455636668 | 6.26E-42 |
| COL5A3 | 0.455020085 | 8.55E-42 |
| ELN | 0.448638469 | 2.05E-40 |
| VCAN | 0.443161739 | 2.98E-39 |
| CTGF | 0.438278007 | 3.11E-38 |
| COL8A2 | 0.435433 | 1.20E-37 |
| COL4A1 | 0.432807754 | 4.11E-37 |
| NID1 | 0.428015061 | 3.79E-36 |
| FMOD | 0.424552847 | 1.85E-35 |
| CD34 | 0.423710312 | 2.71E-35 |
| IGF2 | 0.422213018 | 5.33E-35 |
| P4HA3 | 0.42097933 | 9.30E-35 |
| FLT1 | 0.417981937 | 3.55E-34 |
| TIMP2 | 0.416568176 | 6.65E-34 |
| LOX | 0.413144707 | 3.00E-33 |
| CXCL12 | 0.413020912 | 3.17E-33 |
| ADAMTS5 | 0.411482796 | 6.20E-33 |
| BMP1 | 0.411362929 | 6.53E-33 |
| FN1 | 0.410880622 | 8.05E-33 |
| GJA1 | 0.409481962 | 1.48E-32 |
| ADAMTS4 | 0.409460629 | 1.49E-32 |
| KDR | 0.40716026 | 4.01E-32 |
| COL4A2 | 0.405741922 | 7.35E-32 |
| PDGFRA | 0.573362658 | 1.64E-73 |
| MMP2 | 0.556303579 | 3.98E-68 |
| BGN | 0.550796699 | 1.87E-66 |
| CDH11 | 0.550001309 | 3.25E-66 |
| DCN | 0.548993841 | 6.50E-66 |
| COL6A3 | 0.545527827 | 6.97E-65 |
| TGFB3 | 0.542146999 | 6.87E-64 |
| LUM | 0.541816848 | 8.58E-64 |
| COL5A1 | 0.535493995 | 5.75E-62 |
| COL1A2 | 0.532132456 | 5.18E-61 |
| PDGFRB | 0.531992299 | 5.68E-61 |
| COL12A1 | 0.531393622 | 8.38E-61 |
| NID2 | 0.523090205 | 1.70E-58 |
| COL16A1 | 0.521511304 | 4.60E-58 |
| COL8A1 | 0.520908846 | 6.72E-58 |
| COL5A2 | 0.517618858 | 5.22E-57 |
| THY1 | 0.514727962 | 3.11E-56 |
| COL1A1 | 0.50867651 | 1.23E-54 |
| COL3A1 | 0.50756153 | 2.40E-54 |
| FBN1 | 0.507015566 | 3.33E-54 |
| EFEMP2 | 0.503949976 | 2.06E-53 |
| TIMP3 | 0.502744658 | 4.21E-53 |
| COL6A2 | 0.501949128 | 6.72E-53 |
| SPARC | 0.501897032 | 6.93E-53 |
| COL6A1 | 0.494244355 | 5.91E-51 |
| FBLN1 | 0.48575316 | 7.19E-49 |
| SNAI2 | 0.483746264 | 2.19E-48 |
| POSTN | 0.473157304 | 6.98E-46 |
| COL15A1 | 0.466517325 | 2.34E-44 |
| TEK | 0.465296477 | 4.43E-44 |
| PCOLCE | 0.463231133 | 1.29E-43 |
| ACTA2 | 0.46063424 | 4.93E-43 |
| THBS2 | 0.45846672 | 1.49E-42 |
| CTSK | 0.458425628 | 1.52E-42 |
| FBLN5 | 0.456184697 | 4.75E-42 |
| ADAMTS2 | 0.455636668 | 6.26E-42 |
| COL5A3 | 0.455020085 | 8.55E-42 |
| ELN | 0.448638469 | 2.05E-40 |
| VCAN | 0.443161739 | 2.98E-39 |
| CTGF | 0.438278007 | 3.11E-38 |
| COL8A2 | 0.435433 | 1.20E-37 |
| COL4A1 | 0.432807754 | 4.11E-37 |
| NID1 | 0.428015061 | 3.79E-36 |
| FMOD | 0.424552847 | 1.85E-35 |
| CD34 | 0.423710312 | 2.71E-35 |
| IGF2 | 0.422213018 | 5.33E-35 |
| P4HA3 | 0.42097933 | 9.30E-35 |
| FLT1 | 0.417981937 | 3.55E-34 |
| TIMP2 | 0.416568176 | 6.65E-34 |
| LOX | 0.413144707 | 3.00E-33 |
| CXCL12 | 0.413020912 | 3.17E-33 |
| ADAMTS5 | 0.411482796 | 6.20E-33 |
| BMP1 | 0.411362929 | 6.53E-33 |
| FN1 | 0.410880622 | 8.05E-33 |
| GJA1 | 0.409481962 | 1.48E-32 |
| ADAMTS4 | 0.409460629 | 1.49E-32 |
| KDR | 0.40716026 | 4.01E-32 |
| COL4A2 | 0.405741922 | 7.35E-32 |
| PDGFRA | 0.573362658 | 1.64E-73 |
| MMP2 | 0.556303579 | 3.98E-68 |
| BGN | 0.550796699 | 1.87E-66 |
| CDH11 | 0.550001309 | 3.25E-66 |
| DCN | 0.548993841 | 6.50E-66 |
| COL6A3 | 0.545527827 | 6.97E-65 |
| TGFB3 | 0.542146999 | 6.87E-64 |
| LUM | 0.541816848 | 8.58E-64 |
| COL5A1 | 0.535493995 | 5.75E-62 |
| COL1A2 | 0.532132456 | 5.18E-61 |
| PDGFRB | 0.531992299 | 5.68E-61 |
| COL12A1 | 0.531393622 | 8.38E-61 |
| NID2 | 0.523090205 | 1.70E-58 |
| COL16A1 | 0.521511304 | 4.60E-58 |
| COL8A1 | 0.520908846 | 6.72E-58 |
| COL5A2 | 0.517618858 | 5.22E-57 |
| THY1 | 0.514727962 | 3.11E-56 |
| COL1A1 | 0.50867651 | 1.23E-54 |
| COL3A1 | 0.50756153 | 2.40E-54 |
| FBN1 | 0.507015566 | 3.33E-54 |
| EFEMP2 | 0.503949976 | 2.06E-53 |
| TIMP3 | 0.502744658 | 4.21E-53 |
| COL6A2 | 0.501949128 | 6.72E-53 |
| SPARC | 0.501897032 | 6.93E-53 |
| COL6A1 | 0.494244355 | 5.91E-51 |
| FBLN1 | 0.48575316 | 7.19E-49 |
| SNAI2 | 0.483746264 | 2.19E-48 |
| POSTN | 0.473157304 | 6.98E-46 |
| COL15A1 | 0.466517325 | 2.34E-44 |
| TEK | 0.465296477 | 4.43E-44 |
| PCOLCE | 0.463231133 | 1.29E-43 |
| ACTA2 | 0.46063424 | 4.93E-43 |
| THBS2 | 0.45846672 | 1.49E-42 |
| CTSK | 0.458425628 | 1.52E-42 |
| FBLN5 | 0.456184697 | 4.75E-42 |
| ADAMTS2 | 0.455636668 | 6.26E-42 |
| COL5A3 | 0.455020085 | 8.55E-42 |
| ELN | 0.448638469 | 2.05E-40 |
| VCAN | 0.443161739 | 2.98E-39 |
| CTGF | 0.438278007 | 3.11E-38 |
| COL8A2 | 0.435433 | 1.20E-37 |
| COL4A1 | 0.432807754 | 4.11E-37 |
| NID1 | 0.428015061 | 3.79E-36 |
| FMOD | 0.424552847 | 1.85E-35 |
| CD34 | 0.423710312 | 2.71E-35 |
| IGF2 | 0.422213018 | 5.33E-35 |
| P4HA3 | 0.42097933 | 9.30E-35 |
| FLT1 | 0.417981937 | 3.55E-34 |
| TIMP2 | 0.416568176 | 6.65E-34 |
| LOX | 0.413144707 | 3.00E-33 |
| CXCL12 | 0.413020912 | 3.17E-33 |
| ADAMTS5 | 0.411482796 | 6.20E-33 |
| BMP1 | 0.411362929 | 6.53E-33 |
| FN1 | 0.410880622 | 8.05E-33 |
| GJA1 | 0.409481962 | 1.48E-32 |
| ADAMTS4 | 0.409460629 | 1.49E-32 |
| KDR | 0.40716026 | 4.01E-32 |
| COL4A2 | 0.405741922 | 7.35E-32 |
| PDGFRA | 0.573362658 | 1.64E-73 |
| MMP2 | 0.556303579 | 3.98E-68 |
| BGN | 0.550796699 | 1.87E-66 |
| CDH11 | 0.550001309 | 3.25E-66 |
| DCN | 0.548993841 | 6.50E-66 |
| COL6A3 | 0.545527827 | 6.97E-65 |
| TGFB3 | 0.542146999 | 6.87E-64 |
| LUM | 0.541816848 | 8.58E-64 |
| COL5A1 | 0.535493995 | 5.75E-62 |
| COL1A2 | 0.532132456 | 5.18E-61 |
| PDGFRB | 0.531992299 | 5.68E-61 |
| COL12A1 | 0.531393622 | 8.38E-61 |
| NID2 | 0.523090205 | 1.70E-58 |
| COL16A1 | 0.521511304 | 4.60E-58 |
| COL8A1 | 0.520908846 | 6.72E-58 |
| COL5A2 | 0.517618858 | 5.22E-57 |
| THY1 | 0.514727962 | 3.11E-56 |
| COL1A1 | 0.50867651 | 1.23E-54 |
| COL3A1 | 0.50756153 | 2.40E-54 |
| FBN1 | 0.507015566 | 3.33E-54 |
| EFEMP2 | 0.503949976 | 2.06E-53 |
| TIMP3 | 0.502744658 | 4.21E-53 |
| COL6A2 | 0.501949128 | 6.72E-53 |
| SPARC | 0.501897032 | 6.93E-53 |
| COL6A1 | 0.494244355 | 5.91E-51 |
| FBLN1 | 0.48575316 | 7.19E-49 |
| SNAI2 | 0.483746264 | 2.19E-48 |
| POSTN | 0.473157304 | 6.98E-46 |
| COL15A1 | 0.466517325 | 2.34E-44 |
| TEK | 0.465296477 | 4.43E-44 |
| PCOLCE | 0.463231133 | 1.29E-43 |
| ACTA2 | 0.46063424 | 4.93E-43 |
| THBS2 | 0.45846672 | 1.49E-42 |
| CTSK | 0.458425628 | 1.52E-42 |
| FBLN5 | 0.456184697 | 4.75E-42 |
| ADAMTS2 | 0.455636668 | 6.26E-42 |
| COL5A3 | 0.455020085 | 8.55E-42 |
| ELN | 0.448638469 | 2.05E-40 |
| VCAN | 0.443161739 | 2.98E-39 |
| CTGF | 0.438278007 | 3.11E-38 |
| COL8A2 | 0.435433 | 1.20E-37 |
| COL4A1 | 0.432807754 | 4.11E-37 |
| NID1 | 0.428015061 | 3.79E-36 |
| FMOD | 0.424552847 | 1.85E-35 |
| CD34 | 0.423710312 | 2.71E-35 |
| IGF2 | 0.422213018 | 5.33E-35 |
| P4HA3 | 0.42097933 | 9.30E-35 |
| FLT1 | 0.417981937 | 3.55E-34 |
| TIMP2 | 0.416568176 | 6.65E-34 |
| LOX | 0.413144707 | 3.00E-33 |
| CXCL12 | 0.413020912 | 3.17E-33 |
| ADAMTS5 | 0.411482796 | 6.20E-33 |
| BMP1 | 0.411362929 | 6.53E-33 |
| FN1 | 0.410880622 | 8.05E-33 |
| GJA1 | 0.409481962 | 1.48E-32 |
| ADAMTS4 | 0.409460629 | 1.49E-32 |
| KDR | 0.40716026 | 4.01E-32 |
| COL4A2 | 0.405741922 | 7.35E-32 |
