# Supplementary figures and images for: The Role of Circular RNA CDR1as/ciRS-7 in Regulating Tumor Microenvironment: A Pan-Cancer Analysis
Source: Biomolecules. 2019 Aug 30;9(9):429. doi: 10.3390/biom9090429 (PMC6770779; doi:10.3390/biom9090429)

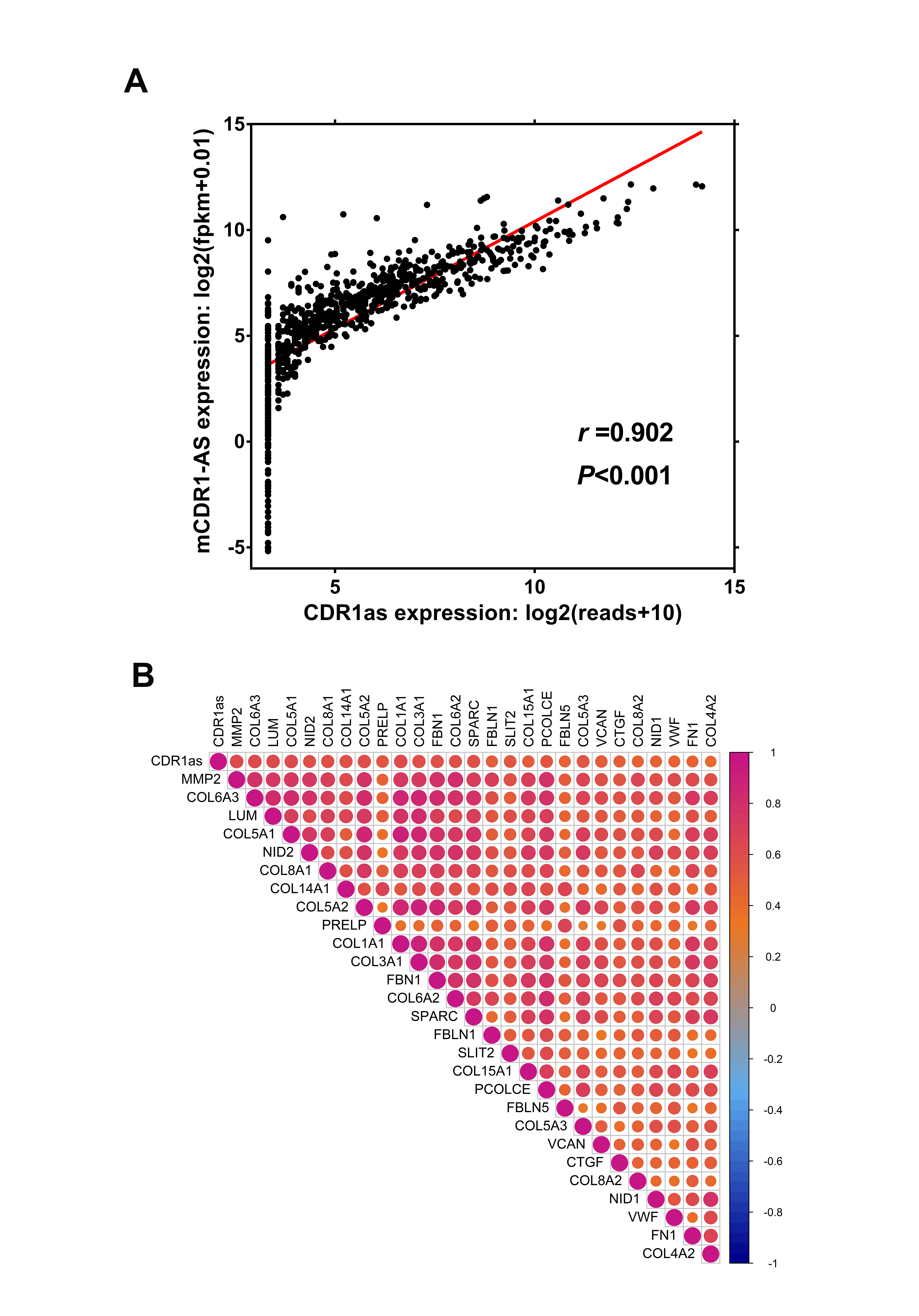

Supplement: Supplementary file 1 [file biomolecules-09-00429-s001.zip › Supplementary File(s)/Supplemental Fig. 1.tif]
